# Genetic variants in a long noncoding RNA related to Sunitinib Resistance predict risk and survival of patients with renal cell carcinoma

**DOI:** 10.1002/cam4.2160

**Published:** 2019-04-30

**Authors:** Qianwei Xing, Ran Li, Aiming Xu, Zhiqiang Qin, Jinyuan Tang, Lei Zhang, Min Tang, Peng Han, Wei Wang, Chao Qin, Mulong Du, Wei Zhang

**Affiliations:** ^1^ Department of Urology The First Affiliated Hospital of Nanjing Medical University Nanjing China; ^2^ Department of Urology Affiliated Hospital of Nantong University Nantong China; ^3^ Department of Urology Surgery Nanjing First Hospital, Nanjing Medical University Nanjing China; ^4^ Department of Urology Jiangsu Province Hospital of TCM, Affiliated Hospital of Nanjing University of TCM Nanjing China; ^5^ Department of Biostatistics, School of Public Health Nanjing Medical University Nanjing China

**Keywords:** LncARSR, renal cell carcinoma, SNPs

## Abstract

**Objective:**

LncARSR (lncRNA Activated in RCC with Sunitinib Resistance, ENST00000424980) is a newly identified lncRNA to promote the sunitinib resistance of renal cell carcinoma (RCC), which may contribute to tumorigenesis and progression. This study aimed to explore the association of lncARSR tagSNPs with the risk and prognosis of RCC.

**Methods:**

In this study, a 2‐stage case‐control study was performed to evaluate the association between 2 tagging SNPs (rs1417080 and rs7859384) and RCC susceptibility. Odds ratios (ORs) and 95% confidence intervals (CIs) were obtained by unconditional logistic regression analyses. Different survival time was estimated by the Kaplan‐Meier method and compared by the Log‐rank test. Hazard ratios (HRs) and their 95% CIs were calculated to determine predictive factors by Cox proportion hazards model.

**Results:**

When combing discovery and validation sets together, rs7859384 was determined to be significantly associated with the decreased RCC risk with all *P* < 0.05 in 4 models (co‐dominant model, additive model, dominant model and recessive model). stratified analyses showed prominent risk effect of SNP rs7859384 GA/GG genotypes was found in clinical subgroups of stage I and stage II (*P* = 0.009, OR = 0.77, 95% CI = 0.64‐0.94) and individuals with clear cell RCC (*P* = 0.014, OR = 0.79, 95% CI = 0.65‐0.95). A protective effect of SNP rs7859384 GA/GG genotypes was observed among individuals with BMI > 24 (*P* = 0.025, OR = 0.74, 95% CI = 0.56‐0.96), without hypertension (*P* = 0.037, OR = 0.79, 95% CI = 0.63‐0.99), without family history of cancer (*P* = 0.048, OR = 0.83, 95% CI = 0.68‐1.00). Survival analyses revealed individuals with GA/GG genotypes had higher survival rate compared with the corresponding AA wild genotypes in the dominant model (log‐rank *P* = 0.005, adjusted HR = 0.34, 95% CI = 0.16‐0.73).

**Conclusion:**

This study suggests that rs7859384 of lncARSR was associated with RCC susceptibility and may act as a prognostic biomarker for patients with RCC.

## 1 INTRODUCTION

An estimated 65,340 Americans were diagnosed with renal malignancy and 14,970 died of the disease in 2018.^1^ Renal cell carcinoma (RCC) is the most common renal malignancy accounting for 90% of the subtypes and approximately 80% of tumors are clear cell renal cell carcinoma (ccRCC).^2^, ^3^ In clinical therapy, surgical resection is just an effective treatment for localized tumor, but the disease still exhibits substantial mortality due to regional or distant metastasis 4 with a characteristic of high resistance toward conventional chemotherapy a radiotherapy.^5^ For advanced RCC patients, receptor of tyrosine kinase (RTK) inhibitors,^6^ such as sunitinib, are regarded as the mainstay of therapeutic options, which has potent anti‐angiogenic effects and direct anti‐tumor activities owing to the inhibition of vascular endothelial growth factor receptor (VEGFR), platelet‐derived growth factor receptor, stem cell growth factor receptor, and FMS‐like tyrosine kinase 3. Despite their efficacy, many RCC patients end up with drug resistance and tumor progression after 6‐15 months of treatment except for those who are inherently refractory to sunitinib therapy.^7^ Recently, a few studies have disclosed the potential molecular biological mechanism of drug resistance such as androgen receptor (AR) phosphorylation,^8^ apoptosis induced by endoplasmic reticulum stress,^9^ sequestration in lysosomes and inhibition of the autophagic flux.^10^ However, few reports focus on genetic biomarkers which might be validated as prognostic factors for patients with sunitinib response.

Long noncoding RNAs (lncRNAs) are transcripts ranging from 200 nt to 100 kb in length with limited protein coding potential.^11^ LncRNAs were once viewed as transcriptional noise, but growing evidence suggests they may play crucial biological roles in transcriptional regulation, cellular development, and RNA modification.^12^ Emerging studies have demonstrated that lncRNAs may be involved in pathogenesis of cancers and they can be prognostic factors referring to tumor initiation and progression. LncARSR (lncRNA Activated in RCC with Sunitinib Resistance, ENST00000424980) is a newly identified lncRNA to promote the sunitinib resistance of RCC by acting as a competing endogenous RNA in the previous study.^13^ Further mechanism reveals that lncARSR can affect the propagation of renal tumor‐initiating cells which may contribute to tumorigenesis, progression, and drug resistance.^14^ In hepatocellular carcinoma, it is established that lncARSR can promote doxorubicin resistance via modulating PTEN‐PI3K/Akt pathway.^15^ Besides, lncARSR may influence hepatic lipogenesis via Akt/SREBP‐1c pathway and contribute to hepatic cholesterol biosynthesis via modulating Akt/SREBP‐2/HMGCR pathway.^16^, ^17^ Therefore, lncARSR could act not only as a therapeutic target to overcome drug resistance but also as a biomarker for improving the prognosis of clinical therapy.

Recently, single nucleotide polymorphisms (SNPs), which could correlate with RCC risk and survival, such as the association of G‐allele of rs231775 in the CTLA‐4 gene with an improved overall survival (OS) in sunitinib‐treated clear cell metastatic RCC patients,^18^ have raised the attention of medical researchers. At this time, according to several studies published to date, SNPs located in the lncRNA locus showed a highly significant association with the susceptibility of a variety of human tumors.^19^, ^20^, ^21^, ^22^ For example, Yan H et al^22^ suggested that rs55829688 polymorphism could increase GAS5 expression by interacting with TP63, which might aggravate the meylosuppression and in turn lead to poor prognosis in acute myeloid leukemia. As a novel long noncoding RNA, lncARSR has been confirmed to participate in the pathophysiological process of cancers, but there are no publications focusing on genetic roles of cancer‐related polymorphisms. Hence, we conducted a hospital‐based cohort study aiming to evaluate the association between lncARSR tagSNPs and RCC risk in a Chinese population.

## 2 MATERIALS AND METHODS

### 2.1 Study population

The present ongoing study was approved by the institutional review board of Nanjing Medical University. Briefly, all subjects were genetically unrelated ethnic Han Chinese recruited coming from different families with no blood relationship. Medical records of all patients were reviewed to ensure no prior history of other cancers or metastasized cancer from other or unknown origins or previously subjected to chemotherapy or radiotherapy. The patients were histopathologically confirmed by 2 pathologists independently and clinical information was obtained, including tumor size, histological type, and tumor metastasis. After signing the written agreement, each of the subjects donated 5 mL venous blood for genomic DNA extraction. More detailed information is presented in previous studies.^23^

### 2.2 SNP selection

**Figure 1:**
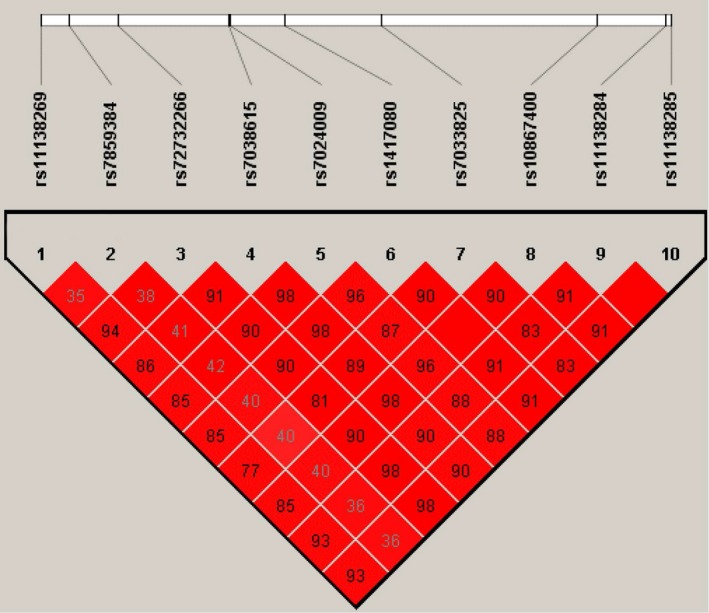
Linkage disequilibrium (LD) plot among the 10 tag‐SNPs in the lncARSR gene (data from 1000 Genome Project database)

Polymorphisms in lncARSR were selected by using genotype data obtained from CHB (Han Chinese in Beijing) and JPT (Japanese in Tokyo) individuals in the 1000 Genome Project database (Phase 1 integrated release 3 March 2012). All the SNPs that had a minor allele frequency >5% and Hardy‐Weinberg equilibrium >0.05 within a 26.5 kb region spanning the lncARSR gene were considered. The identification of the tag‐SNPs was using the pairwise option of the Haploview 4.2 software and an r2 of 0.8 was selected as a threshold for the further analyses. Ultimately, 2 tag‐SNPs (rs1417080 and rs7859384) were selected from all the 10 variant alleles with a mean r2 of 0.945. The identification of the 10 SNPs as well as the LD plot of the SNPs presented by the Haploview 4.2 software is shown in Figure 1.

### 2.3 DNA extraction and polymorphism genotyping

The whole genomic DNA was separated and purified from the peripheral blood leukocyte by proteinase K digestion and phenol‐chloroform extraction according to the manufacturer's instructions (GoldMag Co.Ltd., Xian, China). The genotyping of lncARSR polymorphisms were performed by TaqMan SNP Genotyping Assays (Applied Biosystems, Foster City, CA, USA). The sequences of primer and probe for the single‐nucleotide polymorphism are available on request. Amplification was executed according to the manufacturer's instructions in the 384‐well ABI 7900HT Real‐Time PCR System (Applied Biosystems), and the primers sequence was shown in Table S1. The SDS 2.4 software was used for allelic discrimination. All our procedure of genotyping was carried out in a double‐blind manner. In addition, the random 10% of samples were repeatedly genotyped and the concordance rate was 100%.

### 2.4 Statistical analysis

Using Pearson's chi‐square test for categorical variables and the student's *t*test for continuous variables, differences in the distribution of selected demographic variables and every genotypes between RCC cases and cancer‐free controls were assessed. A goodness‐of‐fit chi‐square test was used to evaluate Hardy‐Weinberg equilibrium (HWE) for all SNP allele frequencies among controls. By using unconditional logistic regression analyses with odds ratios (ORs) and 95% confidence intervals (CIs), the associations between lncRNA SNPs and RCC susceptibility were estimated. Variables of age, sex, body mass index (BMI), smoking status, drinking status, hypertension, diabetes and family history of cancer were as covariates adjusted for the association analysis. Additive, dominant, recessive and co‐dominant genetic models were used to estimate the significance of SNPs. Different survival times were calculated using the Kaplan‐Meier method and compared using the Log‐rank test. Survival time was calculated from the data of RCC diagnosis to the date of death or last follow‐up. Cox proportion hazards models were performed to determine predictive factors of RCC survival by calculating HRs and their 95% CIs. A Cox stepwise regression analysis was performed to determine what factors could be used as an independent factor for gastric cancer prognosis, with *P*  <  0.05 for entering and *P * >  0.10 for removing the model. All statistical analyses were conducted with Statistical Analysis System 9.1.3 software (SAS Institute, Inc, Cary, NC, USA), and the adjusted *P* < 0.05 for 2‐side were considered statistically significant.

## 3 RESULT

### 3.1 Characteristic of study population

In this study, a total of 1002 RCC cases and 1022 cancer‐free controls were recruited in 2 stages, and the demographic and clinical features of individuals in 2 sets were shown in Table S2. There were no significant differences between RCC cases and controls regarding to age, sex, BMI, smoking status, drinking status, and family history of cancer (all *P* > 0.05) while in combined set there were more individuals with hypertension and diabetes in cases than those in controls (both *P* < 0.001). These results imply that hypertension and diabetes might play an important role in the etiology of RCC.

### 3.2 Association between lncARSR polymorphism and risk of RCC

All genotypes distribution of SNPs (iers1417080, rs7859384) among the controls in discovery/validation set was in accordance with HWE (Table 1 and Table 2). In discovery set, rs1417080 in lncARSR was significantly associated with RCC risk (*P* = 0.032, OR = 1.44, 95% CI = 1.03‐2.02 in dominant model) and risk was found in individuals with heterozygote TC genotype (*P* = 0.024, OR = 1.49, 95% CI = 1.05‐2.10 in codominant model). However, in validation set and combined set, no significant association with RCC risk was observed in SNP rs1417080.

**Table 1:** Association of SNP rs1417080 with RCC risk in discovery and validation sets

	Genotype	Case	Control	P (Adjust)	P (Bonferroni correction)	OR (95% CI) (Adjust)		HWE^a^	MAF^b^ (Case/Control)
Discovery set								0.564	0.182/0.148
	TT	226	253			REF			
Co‐dominant model	TC	109	85	0.024	0.048	1.49 (1.05‐2.10)			
	CC	8	9	0.974	1.000	1.02 (0.38‐2.75)			
Additive model				0.065	0.130	1.32 (0.98‐1.78)			
P trend							0.104		
Dominant model	TC+CC	117	94	0.032	0.064	1.44 (1.03‐2.02)			
Recessive model	TT+TC	335	338			REF			
	CC	8	9	0.835	1.000	0.90 (0.33‐2.42)			
Validation set								0.389	0.173/0.192
	TT	439	427			REF			
Co‐dominant model	TC	176	212	0.140	0.280	0.83 (0.65‐1.06)			
	CC	22	21	0.969	1.000	0.99 (0.53‐1.86)			
Additive model				0.251	0.452	0.89 (0.72‐1.09)			
P trend							0.210		
Dominant model	TC+CC	198	233	0.165	0.330	0.84 (0.67‐1.07)			
Recessive model	TT+TC	615	639			REF			
	CC	22	21	0.888	1.000	1.05 (0.56‐1.96)			
Combined set								0.723	0.176/0.177
	TT	665	680			REF			
Co‐dominant model	TC	285	297	0.887	1.000	1.01 (0.83‐1.24)			
	CC	30	30	0.939	1.000	0.98 (0.58‐1.66)			
Additive model				0.945	1.000	1.01 (0.85‐1.19)			
P trend							0.976		
Dominant model	TC+CC	315	327	0.909	1.000	1.01 (0.84‐1.23)			
Recessive model	TT+TC	950	977			REF			
	CC	30	30	0.925	1.000	0.98 (0.58‐1.65)			

a HWE (Hardy Weinberg equilibrium) test among controls.

b MAF (minor allele frequency) between case and control group.

**Table 2:** Association of SNP rs7859384 with RCC risk in discovery and validation sets

	Genotype	Case	Control	P (Adjust)	P (Bonferroni correction)	OR (95% CI) (Adjust)		HWE^a^	MAF^b^ (Case/Control)
Discovery set								0.07	0.343/0.420
	AA	146	126			REF			
Co‐dominant model	GA	164	154	0.416	0.832	0.87 (0.62‐1.22)			
	GG	37	70	0.001	0.002	0.43 (0.26‐0.70)			
Additive model				0.002	0.004	0.70 (0.56‐0.88)			
P trend							0.003		
Dominant model	GA+GG	201	214	0.055	0.110	0.74 (0.54‐1.01)			
Recessive model	AA+GA	310	280			REF			
	GG	37	70	0.001	0.002	0.46 (0.30‐0.72)			
Validation set								0.85	0.365/0.401
	AA	249	235			REF			
Co‐dominant model	GA	312	319	0.512	1	0.92 (0.72‐1.18)			
	GG	77	105	0.029	0.048	0.67 (0.47‐0.96)			
Additive model				0.047	0.094	0.84 (0.72‐1.00)			
P trend							0.108		
Dominant model	GA+GG	389	424	0.202	0.404	0.86 (0.68‐1.08)			
Recessive model	AA+GA	561	554			REF			
	GG	77	105	0.036	0.072	0.71 (0.51‐0.98)			
Combined set								0.349	0.357/0.408
	AA	395	361			REF			
Co‐dominant model	GA	476	473	0.356	0.712	0.91 (0.75‐1.11)			
	GG	114	175	0.000	0.000	0.58 (0.44‐0.77)			
Additive model				0.001	0.002	0.80 (0.70‐0.91)			
P trend	trend						0.001		
Dominant model	GA+GG	590	648	0.040	0.080	0.82 (0.69‐0.99)			
Recessive model	AA+GA	871	834			REF			
	GG	114	175	0.000	0.000	0.62 (0.47‐0.80)			

a HWE (Hardy Weinberg equilibrium) test among controls.

b MAF (minor allele frequency) between case and control group.

When performing the 2 sets analysis of rs7859384, in discovery set we identified that there were less risks in the GG genotype than that in the wild (AA) genotype (*P* = 0.001, OR = 0.43, 95% CI = 0.26‐0.70 in co‐dominant model). Furthermore, the genotypes frequency distributions of SNP rs7859384 in an additive model showed significant difference between cases and controls (*P* = 0.002, OR = 0.70, 95% CI = 0.56‐0.88), and significant effect was also found in the recessive model (*P* = 0.001, OR = 0.46, 95% CI = 0.30‐0.72). Subsequently, in the independent validation set, though rs7859384 in additive model was of marginal difference between cases and controls (*P* = 0.047, OR = 0.84, 95% CI = 0.72‐1.00), in recessive model rs7859384 had significant association with RCC risk (*P* = 0.036, OR = 0.71, 95% CI = 0.51‐0.98). In codominant model, the less risk was consistently related to RCC in the homozygote GG genotype compared with that in the wild genotype (*P* = 0.029, OR = 0.67, 95% CI = 0.96). After combining these 2 stages, a decreased risk of RCC was proven to be associated with the variant allele of rs7859384 in 4 models (all *P* < 0.05).

In addition, stratified analyses of rs7859384 were conducted by clinical and pathological characteristics in the dominant model (Table 3). The prominent risk effect of SNP rs7859384 GA/GG genotypes was noted in clinical subgroups of stage I and stage II (*P* = 0.009, OR = 0.77, 95% CI = 0.64‐0.94). Considering the histology of the tumor, individuals with clear cell RCC had a significant relationship with GA/GG genotypes (*P* = 0.014, OR = 0.79, 95% CI = 0.65‐0.95).

**Table 3:** Stratification analyses between lncARSR rs7859384 polymorphisms and clinicopathologic characteristics in dominant model

Variables	Genotypes		AA vs GA+GG	
	GA+GG,N(%)	AA,N(%)	OR(95% CI)（Adjust）	P (Adjust)
Controls (n = 1009)	648 (64.2)	361 (35.8)	1.00 (reference)	
Cases (n = 985)	590 (59.9)	395 (40.1)	0.82 (0.69‐0.99)	0.040
Clinical stage				
Localized (I/II)	489 (58.5)	347 (41.5)	0.77 (0.64‐0.94)	0.009
Advanced (III/IV)	101 (67.8)	48 (32.2)	1.16 (0.80‐1.68)	0.438
Tumor grade				
Well differentiated (I/II)	426 (59.5)	290 (40.5)	0.82 (0.67‐1.00)	0.051
Moderately differentiated (III)	121 (59.3)	83 (40.7)	0.77 (0.56‐1.06)	0.105
Poorly differentiated (IV)	43 (66.2)	22 (33.8)	1.06 (0.62‐1.82)	0.826
Histology				
Clear cell	480 (58.8)	337 (41.2)	0.79 (0.65‐0.95)	0.014
Others	110 (65.5)	58 (34.5)	1.03 (0.73‐1.46)	0.860

### 3.3 Stratification analyses between lncARSR rs7859384 polymorphisms and clinical risk factors

The effect of lncARSR rs7859384 on RCC occurrence stratified by age, sex, BMI, smoking status, drinking status, hypertension, diabetes, family history of cancer was further investigated (Table 4). A protective effect of SNP rs7859384 GA/GG genotypes was observed among individuals with BMI > 24 (*P* = 0.025, OR = 0.74, 95% CI = 0.56‐0.96), without hypertension (*P* = 0.037, OR = 0.79, 95% CI = 0.63‐0.99), without family history of cancer (*P* = 0.048, OR = 0.83, 95% CI = 0.68‐1.00).

**Table 4:** Stratification analyses between lncARSR rs7859384 polymorphisms and clinical risk factors

Variables	Cases		Controls		OR (95% CI) (Adjust)	P (Adjust)
	GA+GG,N(%)	AA,N(%)	GA+GG,N(%)	AA,N(%)		
Age						
≤57	288 (57.3)	215 (42.7)	377 (65.3)	200 (34.7)	0.76 (0.56‐1.03)	0.073
＞57	302 (62.7)	180 (37.3)	271 (62.7)	161 (37.3)	1.02 (0.78‐1.35)	0.864
Sex						
Male	363 (58.9)	253 (41.1)	433 (64.6)	237 (35.4)	0.80 (0.64‐1.01)	0.058
Female	227 (61.5)	142 (38.5)	215 (63.4)	124 (36.6)	0.89 (0.65‐1.22)	0.468
BMI						
≤24	308 (61.4)	194 (38.6)	344 (63.5)	198 (36.5)	0.91 (0.71‐1.18)	0.497
＞24	282 (58.4)	201 (41.6)	304 (65.1)	163 (34.9)	0.74 (0.56‐0.96)	0.025
Smoking status						
Never	384 (60.1)	255 (39.9)	422 (64.4)	244 (35.6)	0.80 (0.64‐1.01)	0.056
Ever	206 (59.5)	140 (40.5)	206 (63.8)	117 (36.2)	0.91 (0.65‐1.27)	0.583
Drinking status						
Never	439 (60.6)	285 (39.4)	487 (64.8)	264 (35.2)	0.82 (0.66‐1.01)	0.066
Ever	151 (57.9)	110 (42.1)	161 (62.4)	97 (37.6)	0.86 (0.59‐1.24)	0.422
Hypertension						
No	351 (58.2)	252 (41.8)	477 (63.9)	269 (36.1)	0.79 (0.63‐0.99)	0.037
Yes	239 (62.6)	143 (37.4)	171 (65.0)	92 (35.0)	0.90 (0.65‐1.26)	0.552
Diabetes						
No	518 (60.4)	340 (39.6)	611 (64.2)	340 (35.8)	0.83 (0.69‐1.01)	0.066
Yes	72 (56.7)	55 (43.3)	37 (63.8)	21 (36.2)	0.77 (0.39‐1.51)	0.449
Family history of cancer						
No	557 (60.2)	368 (39.8)	606 (64.7)	331 (35.3)	0.83 (0.68‐1.00)	0.048
Yes	33 (55.0)	27 (45.0)	42 (58.3)	30 (41.7)	0.73 (0.33‐1.63)	0.442

### 3.4 Effects of lncARSR rs7859384 on RCC survival

**Figure 2:**
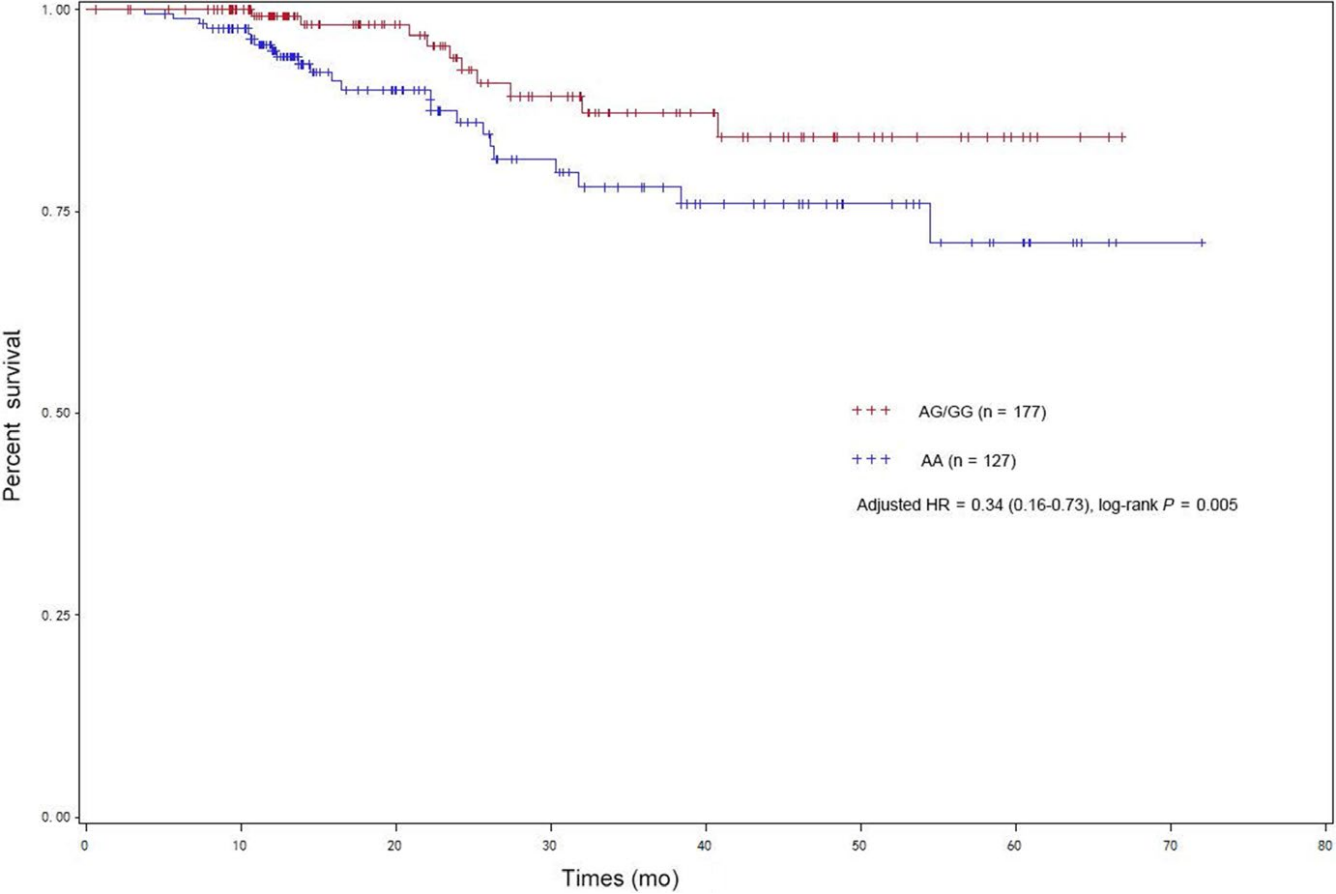
Kaplan‐Meier survival curves for renal cell carcinoma patients with different rs7859384 genotypes in the dominant model

To assess the prognostic value of lncARSR polymorphisms, the clinical follow‐up data on RCC patients' survival were further analyzed. It was reported 311 patients had been followed up and the characteristics and clinical features were showed in the previous study.^23^ However, 304 cases were genotyped for these 311 patients and the median follow‐up time was 19.75 months (minimum‐max, 0.63‐72 months). For rs7859384 of lncARSR, statistically significant association was observed between genotypes and the survival of RCC in the dominant model (log‐rank *P* = 0.005, adjusted HR = 0.34, 95% CI = 0.16‐0.73). As presented in Figure 2, individuals with GA/GG genotypes had higher survival rate compared with the corresponding AA wild genotypes. The stratified analysis implied a significant decreased risk of death among patients of age ≤57 years old, BMI ≤ 24, male, cases without hypertension or diabetes (Table S3). In stepwise Cox proportional hazard analysis for clinical stage, tumor grade and rs7859384 in dominant model, the results indicated that rs7859384 may be an independent prognosis factor with all *P* < 0.05 (Table 5).

**Table 5:** Stepwise Cox regression analysis on RCC‐related survival

Final variable	β	SEM	HR	95% CI	P
Clinical stage	0.68	0.16	1.98	1.45‐2.70	<0.001
Tumor grade	0.59	0.23	1.8	1.15‐2.83	0.009
Dominant model (rs7859384, GA/GG VS AA)	−1.23	0.36	0.29	0.14‐0.59	<0.001

β, regression coefficient.

## 4 DISCUSSION

In this study, we explored the relationship between genetic variants of lncARSR and the risk of RCC in a Chinese population. Our study revealed that rs7859384 variant GA/GG genotypes in lncARSR was associated with a decreased risk of RCC while rs1417080 variant TC/CC genotypes did not show a significant relationship with the risk of RCC. When combining with clinical and histopathological variables, stratified analyses of rs7859384 suggested a significant difference in the distribution of GA/GG genotypes among clinical subgroups of stage1/II and patients with clear cell RCC. Besides, the stratified analysis assessed a protective value of SNP rs7859384 GA/GG genotypes in individuals with BMI > 24, without hypertension, without family history of cancer. In addition, the overall survival analysis noticed the significant association between rs7859384 and survival.

The fact that specific hyperconserved elements in lncRNAs are extensively expressed in tumor cells and are also in some normal cells has been confirmed by whole‐genome sequencing, which are distributed over fragile sites and tumor‐related regions in the chromosomes, suggesting that these elements might play a vital role in the normal development of an individual and that their aberrant expression might lead to cellular malignant transformation.^11^ SNPs which are universally present in lncRNA genes are the most common and genetic variants of concern, and may directly or indirectly result in changes in lncRNA expression levels by various means and then being likely to participate in the genesis and development of cancer.^24^, ^25^, ^26^ Owing to the possibility of being biomarkers for predicting cancer risk, increasing researches focuses on cancer‐related genetic polymorphisms of lncRNAs. To date, SNPs of more than 20 lncRNAs have been identified in human malignant tumors.^27^ For example: HOTAIR, as one of the most closely investigated lncRNAs, polymorphisms of which have been studied in gastric cancer,^19^ prostate cancer,^28^ cervical cancer,^29^ breast cancer,^30^ hepatocellular carcinoma,^31^ oral cancer 32, and lung cancer.^33^ However, there are few reports on SNPs of lncRNAs in RCC. To our knowledge, this study is the first to evaluate the effects of lncRNA polymorphisms on the risk of RCC.

LncARSR was the earliest discovered lncRNA which could be a mediator of sunitinib resistance in RCC by acting as a competing endogenous RNA and confer resistance to sensitive cells by exosome‐mediated transmission.^13^ To explore the potential function of lncARSR relatively in depth, our study performed a comprehensive analysis of the relationship between 2 genetic variants in lncARSR and the susceptibility of RCC, and finally found rs7859384 was associated with the decreased risk in 4 statistical models. Though there is a lack of experimental evidence to elucidate the biological process how the SNPs of lncARSR participate in the tumor initiation and progression, our study provides a feasible basis for further investigation which has been applied to other lncRNAs. There exist several hypotheses documented in the literature. As a potential causal SNP for osteoporosis, rs6426749 was demonstrated to be a distal allele‐specific enhancer regulating expression of a lncRNA (LINC00339) via long‐range chromatin loop formation and rs6426749‐G allele can bind transcription factor TFAP2A, which efficiently elevates the enhancer activity and increases LINC00339 expression.^34^ Yao et al performed in silico analyses to speculate the molecular mechanism underlying the association between rs7958904 and colorectal cancer risk, and the results indicated that rs7958904 G/C variant might participate in colorectal cancer through alteration of HOTAIR secondary structure.^35^ Moreover, rs7958904 polymorphism may affect the binding activity of has‐miR‐615, which can regulate the proliferation, migration, invasion, and apoptosis of various cancers.^36^, ^37^ These studies have initially explored the biological mechanisms of lncRNA SNPs and may be explanations of the way lncARSR SNPs influence the risk of RCC.

After stratified analyses of tumor stage and grade, there was no statistical relationship between lnARSR polymorphism and clinical stage 3/4 of RCC. The result seems to be inconsistent with previous research that lncARSR was first found to promote sunitinib resistance which is a major challenge for advanced RCC. It may be caused by the number of subjects and we'd better enroll more cases and controls for the comprehensive study. However, at stage 1/2 of RCC, rs7859384 GA/GG genotype was preliminarily observed to be markedly decreasing the risk. Interestingly, when controls' genotypes were taken as the reference, a statistically notable association for rs7859384 genotypes and clear cell RCC was identified. RCC is one of the most common malignant neoplasms in the world with diverse histological types including clear cell RCC, papillary RCC, chromophobe RCC, and so on. Yu et al first determined genome‐wide lncRNAs expression patterns in clear cell RCC by microarray providing potential targets for future treatment and novel insights into cancer biology.^38^ There are other reports focusing on the relation of lncRNAs to clear cell RCC,^39^, ^40^ nevertheless, few studies revealed the correlation between lncRNA polymorphisms and tumor histological types. Results of the present study can be plausible considering that 786‐O cell was implemented to investigate the biological process of lncARSR^13^ and 786‐O cell is a kind of clear cell types.

As well as pathological grades and tumor histological types, clinical risk factors have also been well estimated in this study. Intriguingly, our results imply that genetic variants of lncARSR can be protective factors among patients with BMI > 24, without hypertension and without family history of cancer. According to a newly published article,^41^ a person with 5 kg/m^2^ lower BMI has 22% less risk for RCC relative to another person with all other factors equal. Fortunately, GA/GG genotypes contribute to the low risk in population with BMI > 24. It is not contradictory that the result showed no significant correlation between GA/GG genotypes and RCC risk in the population with BMI ≤ 24 taking into account less percentage of risk attributing to lower BMI may not lead to differences among genotypes. However, as shown in Table S3, GA/GG genotypes have a negative correlation with mortality among RCC patients with BMI ≤ 24. It is well known that genetic factors play a critical role in the occurrence of RCC so family history of cancer has been recognized as an exposure risk factor in many cancers. Therefore, it may be interpreted that allele A need to mutate into allele G to take protective effect while it would not happen in the population with the inheritance of tumor family history. Subsequently, protective value of GA/GG genotypes is found to be associated with predisposition to population without hypertension rather than those with hypertension. It is still unknown that arisen of this phenomenon is rooted in some specific genes which can cause hypertension or hypertension which can affect rs7859384 mutation. The increase in risk of RCC due to smoking is approximately the same in males and females.^41^ Graff et al found that type2 diabetes was independently associated with a greater risk of RCC in women but not in men.^42^ On the other hand, a meta‐analysis based on twenty observation studies supports the hypothesis of a negative effect of moderate alcohol consumption on the risk of RCC.^43^ Irrespective of whether the above factors can or cannot impact the risks of RCC, rs7859384 GA/GG genotypes do not act as a protective factor in stratified analyses related to smoking, diabetes, and drinking. In order to understand the role of rs7859384 variants, further experiments are needed to identify the precise mechanisms.

To explore the prognostic role of rs7859384, an overall survival study was established, suggesting GA/GG genotypes can predict a higher survival rate than AA wild genotype. Le et al^14^ demonstrated that lncARSR was up‐regulated in primary renal T‐ICs leading to a poor prognosis of clear cell RCC and knockdown of lncARSR could attenuate the self‐renewal, tumorigenicity, and metastasis of renal T‐ICs. The underlying molecular mechanism may attribute to the fact that variant allele can influence the expression of related lncRNA, which has been proven in the previous studies. Zhang et al proposed that the risk allele rs4321755‐T, in phase with rs4415084‐T, created a GATA3‐bingding motif within an enhancer, resulting in differential GATA3 binding and chromatin accessibility, thereby promoting transcription of MRPS30 and lncRNA RP11‐53O19.1.^44^ Guo et al found that a risk‐associated variant at rs7463708 increases binding of ONECUT2, a novel androgen receptor‐interacting transcription factor, at a distal enhancer that loops to the lncRNA PCAT1 promoter, resulting in up‐regulation of PCAT1 upon prolonged androgen treatment.^45^ Thus, we speculate that expression of lncARSR can be affected by variants at rs7859384 leading to different survival rates of RCC patients.

## 5 CONCLUSION

In summary, this is the first study investigating the epidemiologic evidence on lncRNA SNPs with RCC risks and the related survival in a Chinese population. We found that a SNP rs7859384 of lncARSR had a strong association with RCC susceptibility by 2‐stage case‐control statistical analyses with a relatively large population size. Besides, survival analysis indicated that variant at rs7859384 may contribute to higher overall survival rates. However, more detailed investigations and further experiments on genetic functions will be needed in the future.

## CONFLICT OF INTEREST

We declare that we have no conflict of interest.

## AUTHOR CONTRIBUTIONS

Xing, Li and Xu contributed equally to this work.

## ETHICAL STATEMENT

The present ongoing study was approved by the institutional review board of Nanjing Medical University. Each participant enrolled in this study has provided a written informed consent document.
