# Supplementary material for: Genetic variants in a long noncoding RNA related to Sunitinib Resistance predict risk and survival of patients with renal cell carcinoma
Source: Cancer Med. 2019 Apr 30;8(6):2886–96. doi: 10.1002/cam4.2160 (PMC6558481; doi:10.1002/cam4.2160)
Supplement: Supplementary file 1 [file CAM4-8-2886-s001.docx]

**Supplementary Table 1.** The used primer sequence in the present study

| Primer ID | Sequence (5’-3’) |
| --- | --- |
| rs1417080 (C/T) F | TAGCAGAATATATTAAATTGGAAG |
| rs1417080 (C/T) R | TCATATAAATCTGCATGAGCAT |
| rs7859384 (A/G) F | GTGATTAAATTGTATCATGGCA |
| rs7859384 (A/G) R | CGTTATTCAAGGTCCAGTTATGA |

**Supplementary Table 2**. Demographic characteristics and clinical features of discovery and validation sets among RCC cases patients and control subjects

|  | Discovery set | | P | Validation set | | P | Combined set | | P^a^ |
| --- | --- | --- | --- | --- | --- | --- | --- | --- | --- |
| Characteristics | No.of Cases(%) | No.of Controls(%) |  | No.of Cases(%) | No.of Controls(%) |  | No.of Cases(%) | No.of Controls(%) |  |
| Overall | 355 | 362 |  | 647 | 660 |  | 1002 | 1022 |  |
| Age(years)  (mean±SD) | 57.1±11.8 | 55.6±11.5 | 0.089 | 56.8±12.4 | 57.3±10.2 | 0.442 | 56.9±12.2 | 56.7±10.7 | 0.678 |
| Sex |  |  | 0.120 |  |  | 0.003 |  |  | 0.149 |
| Male | 228(64.2) | 212(58.6) |  | 403(62.3) | 463(70.2) |  | 631(63.0) | 675(66.0) |  |
| Female | 127(35.8) | 150(41.4) |  | 244(37.7) | 197(29.8) |  | 371(37.0) | 347(34.0) |  |
| BMI(kg/m2)(mean±SD) | 24.1±2.9 | 23.7±3.2 | 0.080 | 24.1±2.9 | 23.9±3.3 | 0.432 | 24.1±2.9 | 23.8±3.3 | 0.096 |
| ≤24 | 170(47.9) | 197(54.4) | 0.080 | 338(52.2) | 352(53.3) | 0.693 | 508(50.7) | 549(53.7) | 0.174 |
| >24 | 185(52.1) | 165(45.6) |  | 309(47.8) | 308(46.7) |  | 494(49.3) | 473(46.3) |  |
| Somking status |  |  | 0.172 |  |  | 0.282 |  |  | 0.093 |
| Never | 220(62.0) | 242(66.9) |  | 427(66.0) | 454(68.8) |  | 647(64.6) | 696(68.1) |  |
| Ever | 135(38.0) | 120(33.1) |  | 220(34.0) | 206(31.2) |  | 355(35.4) | 326(31.9) |  |
| Drinking status |  |  | 0.636 |  |  | 0.266 |  |  | 0.570 |
| Never | 249(70.1) | 248(75.1) |  | 486(75.1) | 513(77.7) |  | 735(73.4) | 761(74.5) |  |
| Ever | 106(29.9) | 114(31.5) |  | 161(24.9) | 147(22.3) |  | 267(26.6) | 261(25.5) |  |
| Hypertension |  |  | 0.016 |  |  | <0.001 |  |  | <0.001 |
| No | 220(62.0) | 255(70.4) |  | 393(60.7) | 499(75.6) |  | 613(61.2) | 754(73.8) |  |
| Yes | 135(38.0) | 107(29.6) |  | 254(39.3) | 161(24.4) |  | 389(38.8) | 268(26.2) |  |
| Diabetes |  |  | <0.001 |  |  | <0.001 |  |  | <0.001 |
| No | 310(87.3) | 345(95.3) |  | 564(87.2) | 618(93.6) |  | 874(87.2) | 963(94.2) |  |
| Yes | 45(12.7) | 17(4.7) |  | 83(12.8) | 42(6.4) |  | 128(12.8) | 72(7.0) |  |
| Family history of cancer |  |  |  |  |  |  |  |  |  |
| No | 335(94.4) | 357(98.6) | 0.002 | 607(93.8) | 593(89.8) | 0.009 | 942(94.0) | 950(93.0) | 0.336 |
| Yes | 20(5.6) | 5(1.4) |  | 40(6.2) | 67(10.2) |  | 60(6.0) | 72(7.0) |  |
| Clinical stage |  |  |  |  |  |  |  |  |  |
| I/II | 290(81.7) |  |  | 560(86.6) |  |  | 850(84.8) |  |  |
| III/IV | 65(18.3) |  |  | 87(13.4) |  |  | 152(15.2) |  |  |
| Tumor grade |  |  |  |  |  |  |  |  |  |
| I/II | 238(67.0) |  |  | 491(75.9) |  |  | 729(72.8) |  |  |
| III | 84(23.7) |  |  | 124(19.2) |  |  | 208(20.8) |  |  |
| IV | 33(9.3) |  |  | 32(4.9) |  |  | 65(6.5) |  |  |
| Histology |  |  |  |  |  |  |  |  |  |
| Clear cell | 301(84.8) |  |  | 533(82.4) |  |  | 834(83.2) |  |  |
| Papillary | 8(2.3) |  |  | 31(4.8) |  |  | 39(3.9) |  |  |
| Chromophobe | 17(4.8) |  |  | 38(5.9) |  |  | 55(5.5) |  |  |
| Unclassified | 29(8.2) |  |  | 45(7.0) |  |  | 74(7.4) |  |  |

^a^ Discovery set and validation set merged

**Supplementary Table 3.** Stratified analysis of rs7859384 genotypes associated with RCC patients’ survival

| Variables | rs7859384(Patients/Deaths) | | Log-rank P | Adjusted HR(95%CI)^a^ |
| --- | --- | --- | --- | --- |
|  | GA/GG | AA |  |  |
| Total | 156/21 | 113/14 | 0.005 | 0.34(0.16-0.73) |
| Age (years) |  |  |  |  |
| ≤ 57 | 91/10 | 62/5 | 0.04 | 0.26(0.07-0.98) |
| >57 | 65/11 | 51/9 | 0.09 | 0.48(0.17-1.35) |
| BMI (kg/m2) |  |  |  |  |
| ≤ 24 | 77/11 | 49/9 | 0.002 | 0.19(0.05-0.64) |
| >24 | 79/10 | 64/5 | 0.31 | 0.70(0.22-2.26) |
| Sex |  |  |  |  |
| Male | 101/15 | 72/8 | 0.02 | 0.26(0.09-0.79) |
| Female | 55/6 | 41/6 | 0.09 | 0.09(0.02-0.52) |
| Smoking status |  |  |  |  |
| Never | 99/12 | 73/10 | 0.07 | 0.36(0.14-0.90) |
| Ever | 57/9 | 40/4 | 0.009 | 0.007(0.00-1.01) |
| Drinking status |  |  |  |  |
| Never | 111/15 | 82/9 | 0.07 | 0.43(0.18-1.07) |
| Ever | 45/6 | 31/5 | 0.02 | 0.001(0.00-0.24) |
| Hypertension |  |  |  |  |
| No | 97/11 | 76/7 | 0.007 | 0.27(0.09-0.80) |
| Yes | 59/10 | 37/7 | 0.36 | 0.45(0.11-1.87) |
| Diabetes |  |  |  |  |
| No | 135/19 | 98/12 | 0.01 | 0.35(0.16-0.79) |
| Yes | 21/2 | 15/2 | 0.17 | -b |
| Family history of cancer |  |  |  |  |
| No | 145/20 | 109/11 | 0.07 | 0.38(0.07-0.83) |
| Yes | 11/1 | 4/3 | 0.009 | - b |
| Clinical stage |  |  |  |  |
| Localized (I/II) | 132/4 | 106/5 | 0.003 | 0.16(0.04-0.60) |
| Advanced (III/IV) | 24/17 | 7/9 | 0.04 | 0.43(0.13-1.39) |
| Tumor grade |  |  |  |  |
| Well differentiated (I+II) | 108/3 | 86/4 | 0.03 | 0.16(0.03-0.95) |
| Moderately differentiated (III) | 37/8 | 24/4 | 0.45 | 0.34(0.08-1.51) |
| Poorly differentiated (IV) | 11/10 | 3/6 | 0.02 | 0.04(0.002-1.07) |
| Histology |  |  |  |  |
| Clear cell | 130/11 | 104/10 | 0.005 | 0.28(0.11-0.72) |
| Others | 26/10 | 9/4 | 0.30 | 0.27(0.03-2.87) |

^a^ Adjusted for age, sex, BMI, smoking status, drinking, hypertension, diabetes, family history of cancer, clinical stage, grade and histology in Cox regression Dominant (AG/GG *vs* AA) model.

^b^ HR (hazard ratio) in Cox regression model was not calculated
